# Two dimensional intraoperative transesophageal echocardiography (TEE) allows length-calculation of neo-chordae for mitral valve repair

**DOI:** 10.1186/1749-8090-8-S1-O270

**Published:** 2013-09-11

**Authors:** MH Ammar, A Zandi, H Ebel, H Bigdeli, M Roosta-Azad, M Kamler

**Affiliations:** 1 Cardiac Surgery, Heart Center, Essen, Germany

## Background

The loop technique for mitral valve repair is complicated by accurate estimation of length of neo-chordae in the arrested heart. It was the aim of the study to evaluate the value and accuracy of perioperative dynamic TEE to assess the adequate length of artificial choradae in comparison with the surgeon’s in situ measurements.

## Methods

From July 2010 to April 2013, 95 consecutive patients (mean age, 63 ± 20 years, mean LVEF, 50% ± 15 %), with severe mitral valve regurgitation ( ischemic 17%. PML-prolapse 7.3 %. AML- prolapse 2, 1%. PML and AML-Prolaps 6.3 %. Endocarditis 2.1 %. Barlow 1%. Cleft with AML_+_ PML Prolapse 11,5%. AML. Descaling 5, 2%.) underwent mitral valve repair. Anticipated length of loops was assessed with TEE or with a custom made caliper (Geister Medizintechnik) by measuring the distance between the top of papillary muscle and the cooptation zone of intact leaflets in the end systolic phase or in the arrested heart.

## Results

Overall 30 day hospital mortality was 0 %. Stroke rate was 0%. Respiratory failure rate was 2%. Freedom from reoperation rate was 99 % (one Patient sever MR, ruptured papillary muscle). Follow up Echocardiography showed MR: 0-I° in 98 %, I°-II° in 1% and II°-III° in 1% of patients. Proposed length of neochordae by TEE was 22.1 ± 3 mm, length used by the surgeon was 21.2 ±3 mm.

## Conclusions

For optimization of the length of neo-chordae TEE is a valuable tool to help the surgeon to estimate the correct length in the arrested heart. Discrepancies are due to methological differences between the two methods and TEE values have to be judged by the surgeon in accordance to the local finding in the heart.

